# Fragile X Syndrome

**DOI:** 10.2174/138920211795677886

**Published:** 2011-05

**Authors:** Yingratana McLennan, Jonathan Polussa, Flora Tassone, Randi Hagerman

**Affiliations:** 1 Medical Investigation of Neurodevelopmental Disorders (M.I.N.D.) Institute, University of California Davis Health System, Sacramento, California, USA; 2 Department of Biochemistry and Molecular Medicine, University of California Davis, School of Medicine, Davis, California, USA; 3 Department of Pediatrics, University of California Davis Health System, Sacramento, California, USA

**Keywords:** Fragile X, trinucleotide repeat, Prader-Willi phenotype, obesity, mGluR antagonists.

## Abstract

Recent data from a national survey highlighted a significant difference in obesity rates in young fragile X males (31%) compared to age matched controls (18%). Fragile X syndrome (FXS) is the most common cause of intellectual disability in males and the most common single gene cause of autism. This X-linked disorder is caused by an expansion of a trinucleotide CGG repeat (>200) on the promotor region of the fragile X mental retardation 1 gene (FMR1). As a result, the promotor region often becomes methylated which leads to a deficiency or absence of the FMR1 protein (FMRP). Common characteristics of FXS include mild to severe cognitive impairments in males but less severe cognitive impairment in females. Physical features of FXS include an elongated face, prominent ears, and post-pubertal macroorchidism. Severe obesity in full mutation males is often associated with the Prader-Willi phenotype (PWP) which includes hyperphagia, lack of satiation after meals, and hypogonadism or delayed puberty; however, there is no deletion at 15q11-q13 nor uniparental maternal disomy. Herein, we discuss the molecular mechanisms leading to FXS and the Prader-Willi phenotype with an emphasis on mouse FMR1 knockout studies that have shown the reversal of weight increase through mGluR antagonists. Finally, we review the current medications used in treatment of FXS including the atypical antipsychotics that can lead to weight gain and the research regarding the use of targeted treatments in FXS that will hopefully have a significantly beneficial effect on cognition and behavior without weight gain.

## INTRODUCTION

Obesity is a common problem in the fragile X syndrome (FXS) and we can learn from the molecular overlap between FXS and other obesity syndromes, particularly Prader-Willi syndrome (PWS). A recent national survey from 885 families found similar obesity rates between FXS adults in comparison to typically developing adults [1]. However, body mass index (BMI) data collected from 718 children, with an age <20 years, found male children with FXS had a higher prevalence rate of obesity (31%) when compared to age matched control children (18%) [1]. FXS is the most common cause of inherited intellectual disability (ID) and the most common known single gene cause of autism [2,  3]. It is caused by a CGG expansion greater than 200 repeats in the 5’ untranslated region in the fragile X mental retardation 1 (*FMR1*) gene. The expanded number of repeats can result in either a premutation (55 to 200 repeats) or a full mutation (>200 CGG repeats) status [4, 5]. The full mutation usually leads to methylation of the promoter region so that little or no FMR1 mRNA is produced with lack of the *FMR1* protein (FMRP) [6-8]. In those with FXS the level of cognitive ability and the severity of the physical phenotype correlates with the level of FMRP [9]. The lack of FMRP can lead to accelerated preadolescent growth in FXS but a diminution of the normal pubertal growth spurt [10]. The knockout mouse model of FXS has enhanced growth and obesity that can be reversed by the use of targeted treatments specifically mGluR5 antagonists [11]. 

Finally, in less than 10% of individuals with FXS there is an unusual phenotype associated with severe obesity, hyperphagia, hypogonadism or delayed puberty and termed the Prader-Willi phenotype (PWP), although it is not associated with a deletion of 15q11-q13 nor due to uniparental maternal disomy 15. Instead a lower expression of the *CYFIP1* gene, located in the 15q11-q13 region, encodes for the cytoplasmic FMR1– interacting protein 1 (CYFIP1), a protein that works in concert with FMRP, is associated with the PWP [12]. In addition, those individuals with the PWP and FXS have an autism spectrum disorder (ASD) at a higher frequency than those with FXS alone, without the PWP.

To better understand the association between obesity and FXS in addition to the molecular overlap with other disorders, we will review the function of FMRP in addition to the phenotypic features of FXS and fragile X- associated disorders. Treatment endeavors, particularly those that utilize atypical antipsychotics, can exacerbate the weight gain of individuals with FXS making obesity a significant problem. Therefore, the use of new-targeted treatments in FXS has the potential to reverse or alleviate the obesity in affected individuals as it does in the *FMR1* knockout (KO) mouse.

## MOLECULAR BASIS OF FXS: FMRP DEFICIENCY

FMRP is expressed in many tissues but is mainly concentrated in the neuronal cells in the brain and testes [13, 14]. FMRP is an RNA-binding protein that selectively binds to as much as 4% of all mRNA in mammalian brains [15]. While FMRP deficiency is the cause of FXS, one preliminary report shows a deficiency of FMRP in the brains of individuals with neuropsychiatric disorders that do not have an *FMR1* mutation [19]. Post-mortem brain tissue from the lateral cerebella of controls compared to subjects with psychiatric disorders revealed FMRP was reduced by 78% in the brains of those with schizophrenia, 68% in major depression, and 60% in brains of those with bipolar disorder as compared to control brains [19].

FMRP is involved in multiple roles including the transport of mRNAs to the synapses [16, 17] and repression of mRNA translation (perhaps for both initiation and elongation phases) [3, 4]. Napoli and colleagues [18] demonstrated that FMRP binds to CYFIP1 (Cytoplasmic FMRP Interacting Protein 1) and this complex binds to eIF4E, a translation initiation factor involved in the directing of ribosomes to the cap structure of mRNAs. The resulting eIF4E-CYFIP1-FMRP complex is present at synapses. Synaptic stimulation leads to the release of CYFIP1 from eIF4E, allowing translation to occur [18]. 

The *FMR1* gene is located on chromosome Xq27.3 with the mature mRNA composed of 17 coding exons spanning 38kb. Several highly conserved regions including the NLS domain (exons 5-6), NES domain (exon 14), the first KH1 domain (exons 7-9), the second KH2 domain (exons 9-11), and the RGG (exon 15) are found [15]. Amino acid sequences encoding for the nuclear export signal (NES) and nuclear localization signal (NLS) demonstrate that FMRP travels back and forth between the nucleus and cytoplasm. However, the roles of FMRP within the nucleocytoplasmic space are not fully understood, although it is known that FMRP binds and transports mRNAs to the synapse. FMRP can also stabilize the mRNAs (PSD-95 mRNA) or enhance the degradation of mRNAs (Nfxf1 mRNA) [20] and appears to shape the pattern of mRNA regulation throughout development [20].

Most of the information known about FMRP involves the interactions between the local dendritic mRNA bindings through attachments identified by the K homology (KH) domains and a RGG box, composed of arginine and glycine residues. Proteins with these types of motifs have been shown to be RNA-binding proteins. The significance of these attachments between polyribosomes and FMRP was confirmed in a case study where molecular analysis revealed normal levels of FMRP in an individual with a point mutation in 1 of the 2 KH domains, preventing proper bindings and thus the assemble of RNP complexes [21, 22].

Complementary DNA (cDNA) of most of the associated mRNAs was found using protein complex immunoprecipitation (Co-IP) techniques on FMRP and then identified through microarrays [23]. Many FMRP interacting mRNAs have yet to be discovered but those that have been identified as targets of FMRP include Arc (activity-regulated cytoskeleton-associated protein), CamKII alpha (calcium/ calmodulin-dependent protein kinase), eEF1A (elongation factor 1a), GluR1/2, Sapap3/4 (postsynaptic scaffolding proteins; bind to PSD-95), RGS5 (regulator of G-protein signaling 5), and GABA_A_ [23]. Additional research involving Co-IP or in-vitro methods found APP (amyloid beta (A4) precursor protein), *FMR1*, MAP1B (microtubule-associated protein 1B), PSD-95, and Sema3F (semaphorin 3F) binding to FMRP through a G-quartet-like structure that has been shown to interact with the RGG box *in vitro* [15]. Results from several studies show that most of the mRNA targets are found to be mGluR stimulated (with the exception of Sema3F) and localized within the dendrites (with the exception of APP) [15]. Together with FMRP, microRNAs, ribosomes, and proteins form mRNP (messenger riboucleoprotein) complexes that are transported down to the dendritic spines in anticipation of an action potential to signal the start of protein synthesis [24]. These mRNP complexes are vital in post-transcriptional processes because of their specificity for binding and interacting with recognizable transcripts only, which suggest the flow of transcripts is not as generic as previously thought, but rather a highly precise mechanism performed through central gene expression [25].

The necessity for local translation to take place in the dendritic spines through the binding of these mRNA targets is vital for synaptic plasticity. This ability to respond quickly to stimuli by adapting in synaptic strength is crucial for evolutionary conserved processes such learning and memory [11]. This mechanism requires the presence of protein machinery proximal to the distal synaptic connections, as opposed to the perinucleur regions of the cytoplasm, to quickly and efficiently respond to incoming signals and remodel accordingly. Visualizations from *FMR1* KO mice and FXS human tissues reveal an increased density of immature elongated spines located on the dendrite [26]. Dendritic spines are used to increase the area of contact between other neurons and support the electrical signals from action potentials. Healthy spines in WT mice reveal bulbous heads at the end of necks that appear plump with full knobs enriched with AMPA (alpha-amino-3-hydroxy-5-methyl-4-isoxazolepropionic acid) receptors, GluR (glutamate receptors), and NMDA (N-methyl-D-aspartate) receptors. The dendrites of FXS look immature due to the loss of these AMPA and NMDA receptors. Furthermore these immature spines show a reduction in synaptic vesicles docking at the presynaptic active zone and a smaller postsynaptic density [27]. *FMR1* KO mice show the same immature spine profiles and have learning and memory problems due to a decrease in synaptic plasticity [28]. 

FMRP is believed to affect several pathways including the mGluR signaling pathway. Glutamate is the principal excitatory neurotransmitter and the dynamics of fluctuations can result in two key mechanisms for learning: Long Term Depression (LTD) and Long Term Potentiation (LTP). LTD is a form of synaptic plasticity that is regulated by activation of mGluRs that leads to an internalization of AMPA receptors. The mGluR hypothesis, initially proposed in 2004 [29], suggests that the disruption of the role of FMRP as suppressor of regulation on the mGluRs leads to the over internalization of AMPA receptors. *FMR1* KO studies demonstrated activation of mGluR in the hippocampal field CA1 and in cultured hippocampal neurons leading to exaggerated LTD when compared to WT mice [30, 31]. The mGluR hypothesis is also supported by the rescue of audiogenic and limbic seizures, characteristic of FXS [32] in *FMR1* KO mice treated with MPEP, a selective noncompetitive antagonist of mGluR. Interestingly, the enhanced weight gain in *FMR1* KO mouse which is 30% increased compared to wild type [33] is rescued when the *FMR1* KO mice are crossed with an mGluR5 deficient mouse [33].

A recent double KO study brought attention to cell signaling through additional G protein coupled receptors known as regulator of G-protein signaling (RGS) proteins. The double knockout (*FMR1* / RGS4) mice were able to rescue a subset of molecular abnormalities seen in FXS, including the restoration of GABA expression and the normalization of PSD-95 mRNA levels [34]. In addition, these double knockout mice had normal weight gain and positive social outcomes, displayed from two independent socialization tests with no statistical significance when compared to WT mice. In comparison, the *FMR1* KO had a 13-17% increase of weight when compared to the other groups and social avoidance behavior [34]. However, macroorchidism and hyperactivity were not affected by the elimination of the RGS4 protein and the reduction of mGluR signaling. Previous studies of mGluR5 antagonists showed no rescue of macroorchidism in the KO mouse [11]. 

Ampakines, positive AMPA receptor modulators, are another form of treatment that can be given to individuals with FXS but no significant effect was seen in a controlled trial [35]. Ampakines are thought to enhance memory because of interactions with BDNF (Brain Derived Neurotrophic Factor) [35], which has been shown to correct hippocampal LTP in some disorders impacted by memory impairment. BDNF levels are not reduced in *FMR1* KO mice; however, it is thought to be mis-localized in hippocampal and neocortical neurons [36]. Besides the glutamargeic system, AMPA receptors are impacted from the dopamergic system as well. Hyperactivity, impulse control, and attention deficits are characteristic of children with FXS and are thought to arise from dysfunction in frontal-subcortical circuits due to a reduced dopaminergic drive [37]. In one national survey that involved data collected from 976 full mutation males and 259 full mutation females; 80% of parents indicated their child had been co-diagnosed or treated for attention problems [38]. 

Another neurotransmitter impacted in FXS is GABA, the inhibitory neurotransmitter of the central nervous system, which, in conjunction with glutamate, helps weaken selective synaptic strength [39]. The GABA hypothesis of FXS hypothesizes an altered gamma-aminobutyric (GABA) receptor signal [11] that affects neuronal synaptic plasticity and leads to developmental difficulties. The mRNA coding for GABA receptor subunits are targeted by FMRP in the cytoplasm [11] and found, in addition to other elements in the GABA signaling pathway, to be down regulated in individuals with FXS [40]. The GABA_A_ receptor proteins appear to be down regulated in the absence of FMRP in the fragile X knockout mice [40, 41]. Recently, the testing of targeted treatments of the GABA pathway has begun in an effort to up-regulate the pathway to near normal levels. These include drugs such as Arbaclofen, a GABA_B_ agonist, and Ganaxolone, a GABA_A_ agonist [11, 42]. Trials of these drugs in animals and humans with FXS are ongoing.

Since FMRP is thought to function as a suppressor of translation, the absence of FMRP manifests as an up-regulation of several proteins since there is a lack of suppression in FXS [43]. This absence of inhibition from FMRP translates into dysfunction of synaptic plasticity and results in several cognitive impairments and behavioral problems in FXS. 

Thus, FMRP regulates local dendritic mRNA translation through binding interactions between various mRNP complexes that suppresses protein synthesis and also mGluR pathways. This results in an over internalization of AMPA receptors which leads to a magnification of LTD: one of the two key processes that facilitates synaptic plasticity to augment learning and memory mechanisms. In addition, interactions between FMRP and neurotransmitter systems have revealed the reversal of increased weight phenotypes through mGluR antagonists.

## THE PHENOTYPE OF FXS AND THE PRADER-WILLI PHENOTYPE

Classic physical features often seen in adults with FXS include an elongated face with a prominent forehead and macroorchidism, 2 or 3 times the normal size by mid-adolescence. In addition, changes in elastin fibers in connective tissue relate to abnormalities, such as prominent ears, soft skin, flat feet, and hyperextensible finger joints. Medical problems often seen in males with FXS include strabismus (8 to 36%), seizures (20%), and otitis media (85%) [44]. 

The behavioral phenotype of FXS includes significant anxiety, attention deficit hyperactivity disorder (ADHD), and hyperarousal to sensory stimuli [45-47]. Surveys of individuals with FXS show there is enhanced food selectivity in the population as well, with the most common type of food sensitivity due to texture (as compared to generalized food refusal or food selectivity due to type of food) [1]. In addition, these patients tend to perseverate on topics sometimes meeting criteria for obsessive-compulsive disorder (OCD) [45]. When their obsessions are focused on food then overeating is a problem and this is commonly seen in the PWP. Although cases involving the removal of food items from the garbage, hoarding or raiding the refrigerator at night are less common in FXS as compared to PWS. However, night eating is often seen in PWP because sleep disturbances are common in FXS and they often are up at night and wander in the house [48].

Ten percent of individuals with FXS may have PWP with severe obesity. The PWP of FXS was first recognized in cases characterized physically by their short stature and enhanced weight [49]. Their distinctive physical phenotype is often misclassified as Prader-Willi syndrome (PWS), a disorder cause by a paternally derived deletion in the 15q11-q13 region or uniparental maternal disomy, but further DNA analysis confirms no abnormalities for both copies of the 15q chromosomes in PWP individuals [12].

**Fig. (1). A, B, & C Prader-Willi Phenotype:**
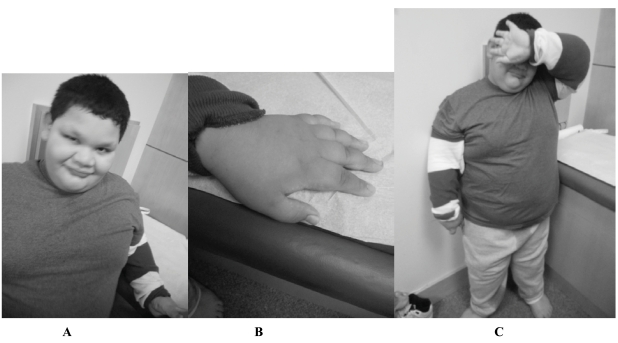
A 9 year, 8-month-old full mutation fragile X boy with the Prader-Willi phenotype. He was referred to the Medical Genetics Clinic where Prader-Willi syndrome was ruled out through methylation testing. A karyotype showed a normal male, 46, XY. Fragile X testing was done and came back abnormal with mosaicism for a full mutation and a low level 105 repeat premutation. Notice the round face and prominent ears (Fig. **1A**), short fingers (Fig. **1B**), and truncal obesity (Fig. **1C**).

Several separate studies reported within the last two decades describe the unusual obesity trend noted in the PWP [50, 51]. These PWP individuals were described as having a lack of satiation and hyperphagia with onset in the first decade of life leading to truncal obesity with fat deposits settling primarily around the torso and abdomen. Among the individuals first described with PWP [50], 2 were first classified as having PWS before cytogenetic testing was available and later nullified and confirmed to have FXS instead. This sub-phenotype of FXS was further distinguished by a deviation away from the traditional Martin-Bell phenotypic features. Most of these individuals with the PWP were described as having a full round face, areas of hypopigmentation, and microorchidism instead of the typical long face, prominent ears and macroorchidism associated with conventional FXS. In addition, they displayed traits that resembled PWS such as severe obesity (>2SD) and short hands/ feet (Fig. **1**). However, none of the individuals with PWP reported having severe hypotonia problems leading to failure to thrive during infancy, which is commonly noted in PWS [12, 49, 50]. Another 13 cases with the PWP and confirmed FXS were reported in 2007 [12]. All 13 had hyperphagia with an average onset at 4.7 years. Consistent with the previous reports of PWP, the actual onset of hyperphagia presents later in childhood in comparison to children with PWS. Another distinction was found in eating habits with PWP regarding binge eating to the point of vomiting, whereas those with PWS have decreased emesis [12]. Precautions such as locking up refrigerators were undertaken in occasional cases when individuals would devour anything available including raw meat and whole sticks of butter due to their lack of satiation while eating (Table **1**). 

Hypogenitalism and hypogonadism was noted in 6 out of the 13 cases with PWP reported by Nowicki *et al*. [12]. Those with hypogonadism in the PWP of FXS eventually developed large testes during puberty consistent with FXS in our experience. None of the 13 cases seen by Nowicki *et al.* [12] reported a short stature with average height at the 73rd percentile. Of the 13 PWP individuals identified by Nowicki *et al.* [12], 8 had IQ data available and FSIQ ranged between 36 and 49 with one exception of a mosaic male who had a FSIQ of 75. These data are consistent with previous reports of cognitive abilities in those with FXS [52]. Interestingly, a higher frequency of autism spectrum disorders was reported in those with the PWP compared to those with FXS without the PWP [12].

Earlier case reports of PWP in FXS hypothesized that features such as the short stature and obesity was a result of hypothalamic dysfunction [49]. Further endocrine studies in FXS have demonstrated hypothalamic-pituitary-adrenal axis dysfunction. In the KO mouse model, there is an elevated level of corticosterone as well as other stress hormones [53]. Cortisol elevations have also been seen in those with FXS particularly at bedtime compared to controls [54].

## OXIDATIVE STRESS IN FXS

Individuals with FXS have a higher level of mitochondrial oxidative stress due to the absence of FMRP [55]. There is an increase in the production of reactive oxygen species [55] as a byproduct of normal cellular function. These reactive oxygen species may affect neural plasticity in the brain as in other syndromes including autism [56, 57]. One of the many enzymes responsible for reducing the negative effects of reactive oxygen species is NADH-oxidase. In the central nervous system, normal functions of NADH-oxidase may play a vital role in neuronal signaling and memory, while the overproduction of reactive oxygen species can lead to neurotoxicity and neurodegeneration [58]. In FXS there is also a deficit in the antioxidant system to control reactive oxygen species and this appears to be related to an alteration in the glutathione system [55].

The over-production and lack of control of reactive oxygen species have lead to research to treat FXS individuals with anti-oxidants. High doses of lipid-soluble antioxidants, especially alpha-tocopherol, have been experimentally shown to reverse hyperactivity, anxiety, and corticosterone elevation in the KO mice [59]. Melatonin, which is an antioxidant in addition to a sleep hormone, has been found to have effects similar to alpha-tocopherol in the KO mice with improvement in synaptic connections and behavioral measures [60]. Recently mitochondrial problems have also been documented in those with the premutation with or without the fragile X-associated tremor ataxia syndrome (FXTAS) [61]. Such studies suggest the need to further study the benefit of antioxidants in those with the premutation and the full mutation.

Obesity and hyperphagia features in PWS have been linked with a 187kb microdeletion on chromosome 15q11-q13 for a non-coding region associated with small nucleolar RNA (snoRNA), including snoRNA HBII-85, that modify other small nuclear RNAs (snRNA) by directing site-specific 2'-O- methylation of substrate RNAs [62]. While there is some early evidence of alternative splicing and variations of FMRP isoforms affecting synaptic plasticity [63], studies have yet to determine if these isoforms also play a role in other phenotypic domains such as the characteristics seen in the PWP [12].

## AUTISM

Autism is a common problem in those with FXS and occurs in approximately 30% of males with FXS. An additional 30% of males who did not meet criteria for autism are diagnosed with ASD [64, 65]. In those individuals with the PWP the rate of autism was 54% which was higher than those without the PWP [12]. Screening for the *FMR1* mutation in those affected by autism or ASD is medically indicated and the rates can be as high as 6% positive for FXS [3]. In a recent study of those with FXS and autism and in those with autism secondary of a 15q duplication, there was abnormal expression of GPR155 (G protein coupled receptor 155) in both groups which is a gene regulated by *CYFIP1* [66]. This may be part of the link between the PWP and autism. 

It has been shown that those individuals diagnosed with PWS are also at increased risk for autism [67]. Individuals with both diagnoses show a high level of similarity with regard to overall repetitive and ritualistic behavior [68]. Studies are now showing that behaviors are similar enough to be predictive of the family of disorders at 15q11-q13 with high accuracy [69]. In PWS, the first microdeletion between BP1 to BP3, known as the type 1 deletion, includes *NIPA1*, *NIPA2*, and *CYFIP1* and these genes are suspected to have the greatest impact in cognitive and behavioral manifestations in PWS [70, 71]. Individuals with Prader-Willi type 1 deletions have more severe cognitive deficits and were more prone to obsessive compulsive disorders and poor adaptive behavior scores than those who had type 2 deletions or uniparental maternal disomy. Furthermore, they showed greater self-injurious behavior and deficits in adaptive behavior [70]. This is reminiscent of the more severe behavioral problems seen in those with the PWP of FXS who have lowered *CYFIP1* compared to those with FXS without the PWP [12]. 

While autism is a heterogeneous disorder with multiple genetic and perhaps environmental factors contributing to the etiology; the *CYFIP1* gene continues to show a molecular link between PWS, PWP in FXS, and autism. Evidence shows that *CYFIP1* dysregulation occurs in almost all individuals with FXS but is down-regulated in the PWP subpopulation. The dysregulation of *CYFIP1* was further confirmed in another *in vivo* study through the dysregulation in ASD of two other genes, *JAKMIP1* and *GPR155*, downstream of *CYFIP1* [66]. Nowicki *et al*. [12] reported that the mRNA levels for *CYFIP1* in the PWP were significantly reduced compared to those with FXS and to controls. The reduced expression of *CYFIP1* mRNA may provide an obstacle in the neuronal plasticity of the brain resulting in the manifestation of higher rates of autism due to the dysregulation of neuronal connections. Model studies of *CYFIP1* mutants implicate that *CYFIP1* is involved in several processes such as in axonal path finding and growth and synaptic morphology at the neuromuscular junction [72]. In addition to the local translation of protein, which is regulated by FMRP, the physical remodeling of the cytoskeleton must be able to adapt in response to extracellular signals as well for normal neuronal morphogenesis and connectivity to occur [72]. The RhoGTPase pathway has been shown to control the actin reorganization in the cytoskeleton [73]. *CYFIP1* has been shown to interact with the RhoGTPase systems and FMRP through Rac1, a RhoGTPase family member, in Drosophila [72]. The loss of FMRP *in vitro* and *in vivo* causes synaptic aberrations that are remarkably similar to those observed in mutants affecting the Rac1 signaling pathways [28, 74]. In Drosophila, *CYFIP1* was found to be a crucial effector of dRac1 and provided a link between FMRP translation and the RhoGTPase actin reorganization processes in a unique pathway to modulate neuronal morphogenesis. 

## TREATMENT OF FXS

Current treatments of FXS include the use of stimulants, selective serotonin reuptake inhibitors (SSRIs), atypical antipsychotics and alpha agonists [75]. ADHD symptoms are seen in the majority of boys and about 30% of girls with FXS [75]. Although stimulants usually decrease the appetite, the atypical antipsychotics usually increase the appetite and these medications are commonly used in FXS to stabilize mood and decrease aggression. In a clinical survey of a large population of children and adults with FXS, 80% responded to > 1 ayptical antipsychotic [76]. Risperidone and aripiprazole are most commonly used with advertising suggesting that aripiprazole has less weight gain associated with use. However, even though low doses of aripiprazole are typically used in FXS because higher doses can be associated with significant agitation, weight gain is still commonly seen. Perhaps the genetic predisposition to obesity in FXS will lead to more frequent weight gain with the use of these medications compared to other individuals without the absence or deficiency of FMRP.

The new targeted treatments for FXS that are focused on reversing the neurobiological problems associated with a lack of FMRP have not caused obesity. In fact, in the mouse model, treatment can rescue the excessive weight gain [11, 33]. These targeted treatments include mGluR5 antagonists that can rescue the dendritic spine deficits, seizures, weight gain, cognitive deficits and behavioral problems in the KO mouse [11, 33]. Human studies have been initiated with benefits seen behaviorally and in prepulse inhibition (PPI) in adult subjects with FXS after the use of one dose of fenobam, an mGluR5 antagonist [77]. Recently, the European trial has published data regarding the use of AFQ056, another mGluR5 antagonist, in adults with FXS which demonstrated efficacy in behavioral measures for those with a full mutation only, but not in those who were mosaic [78]. The preliminary data on the use of Arbaclofen, a GABA_B_ agonist that lowers glutamate at the synapse, demonstrate efficacy in children and adults with FXS and autism or FXS and social deficits rated by the Aberrant Behavioral Checklist [79]. This targeted treatment is also currently being studied in children and adults with idiopathic autism without FXS although the results regarding weight changes are not yet available. 

A new targeted treatment found to be effective in rescuing the dendritic abnormalities in the KO mouse when given after birth is minocycline. This medication can lower the matrix metalloproteinase 9 (MMP9) levels, which are elevated in the KO mouse and strengthen and mature synaptic connections [80]. Since this medication is used clinically to treat acne and readily available by prescription, it has been used by many families and a survey of benefits and side effects have demonstrated that approximately 70% of families observed benefits [81]. The side effects were mainly gastrointestinal including loss of appetite, loose stools or GI intolerance of the medication. An open trial by minocycline in adolescent and young adults with FXS also demonstrated efficacy [82] and currently a controlled trial in children and adolescents with FXS is taking place. We do not yet know whether minocycline will lead to weight loss and whether the appetite suppressing effects are related to the direct effect on the stomach by minocycline or to a neurochemical change. Clinically, the use of a probiotic has helped to decrease the frequency of loose stools because it replaces the GI bacteria with *Lactobacillus* species to overcome the negative effects of minocycline’s influence on reducing GI flora [81].

## CONCLUSIONS

The field of FXS is exciting because of expanding knowledge in molecular biology and the advances in the use of targeted treatments to reverse the neurobiological abnormalities in affected individuals. Further research will clarify why there is enhanced weight gain in both the animal models and in those affected with FXS. The PWP is an interesting bridge between PWS and FXS and further elucidation of the hormonal and molecular dysfunction in PWP will lead to new treatments that may be of benefit to both FXS and PWS.

## Figures and Tables

**Table 1:** Differences and Similarities between Prader-Willi Phenotype, Prader-Willi Syndrome, and Fragile X Syndrome

	Facial Features	Physical Features	Behavioral Features	Macroorchidism	Other
Prader-Willi Phenotype[12]	Round shaped face; almond shaped eyes; ears may or may not be prominent	Obesity; delayed puberty; small penis; hypotonia	Developmental delay; food related behavior problems; hyperphagia; transitions difficult; emesis is common; perseverative speech; behavior problems; hand flapping; poor eye contact; autism or ASD; OCD	Yes, with puberty	
Prader-Willi Syndrome [83]	Almond shaped eyes; strabismus; thin upper lip; downturned corners of mouth; viscous saliva; enamel hypoplasia	Short stature; obesity; osteoporosis; small hands/feet; hypopigmented hair, nails, and skin; frontal hair upsweep	Learning disabilities; hyperphagia; skin/anal picking; food related behavior problems; transitions difficult; stubbornness; perseverative speech; OCD	No	Unusual jigsaw puzzle skill; high pain tolerance
Fragile X Syndrome [84]	Large, prominent ears; long, narrow face; puffiness around eyes; narrow palpebral fissures; large head relative to body; epicanthal folds; strabismus; hypotonia; long and narrow eye openings; prominent jaw; high arched palate	Flat feet; hand calluses; single palmar crease; double jointed thumbs; hypotonia	Tantrums; hyperactivity; anxiety; irritability; autism or ASD; hand flapping and poor eye contact; hyperarousal	Yes with puberty; average range: 40-60 ml	
